# Enabling room temperature ferromagnetism in monolayer MoS_2_ via in situ iron-doping

**DOI:** 10.1038/s41467-020-15877-7

**Published:** 2020-04-27

**Authors:** Shichen Fu, Kyungnam Kang, Kamran Shayan, Anthony Yoshimura, Siamak Dadras, Xiaotian Wang, Lihua Zhang, Siwei Chen, Na Liu, Apoorv Jindal, Xiangzhi Li, Abhay N. Pasupathy, A. Nick Vamivakas, Vincent Meunier, Stefan Strauf, Eui-Hyeok Yang

**Affiliations:** 1 Department of Mechanical Engineering, Stevens Institute of Technology, Hoboken, NJ 07030 USA; 2 Department of Physics, Stevens Institute of Technology, Hoboken, NJ 07030 USA; 3 Center for Quantum Science and Engineering, Stevens Institute of Technology, Hoboken, NJ 07030 USA; 4 Institute of Optics, University of Rochester, Rochester, NY 14627 USA; 5 Department of Physics, Applied Physics, and Astronomy, Rensselaer Polytechnic Institute, Troy, NY 12180 USA; 6 Center of Functional Nanomaterials, Brookhaven National Laboratory, Upton, NY 11973-5000 USA; 7 Department of Physics, Columbia University, New York, NY 10027 USA

**Keywords:** Ferromagnetism, Spintronics, Magnetic properties and materials, Two-dimensional materials

## Abstract

Two-dimensional semiconductors, including transition metal dichalcogenides, are of interest in electronics and photonics but remain nonmagnetic in their intrinsic form. Previous efforts to form two-dimensional dilute magnetic semiconductors utilized extrinsic doping techniques or bulk crystal growth, detrimentally affecting uniformity, scalability, or Curie temperature. Here, we demonstrate an in situ substitutional doping of Fe atoms into MoS_2_ monolayers in the chemical vapor deposition growth. The iron atoms substitute molybdenum sites in MoS_2_ crystals, as confirmed by transmission electron microscopy and Raman signatures. We uncover an Fe-related spectral transition of Fe:MoS_2_ monolayers that appears at 2.28 eV above the pristine bandgap and displays pronounced ferromagnetic hysteresis. The microscopic origin is further corroborated by density functional theory calculations of dipole-allowed transitions in Fe:MoS_2_. Using spatially integrating magnetization measurements and spatially resolving nitrogen-vacancy center magnetometry, we show that Fe:MoS_2_ monolayers remain magnetized even at ambient conditions, manifesting ferromagnetism at room temperature.

## Abstract (web summary)

Ferromagnetism with a Curie temperature above room temperature in 2D materials is highly desirable for practical spintronics applications. Here, the authors demonstrate such phenomenon in monolayer MoS_2_ via in situ iron-doping and measured local magnetic field strength up to 0.5 ± 0.1 mT.

## Introduction

Spintronics requires active control of the spin degrees of freedom in solid-state systems. Dilute magnetic semiconductors (DMSs) are materials in which ferromagnetism and semiconducting properties coexist, offering the capability of manipulating spin-polarized charge carriers for information storage applications toward spintronics. Doping of transition metal elements, such as iron (Fe) and manganese (Mn), into nonmagnetic bulk semiconductors is known to facilitate the formation of DMSs, such as p-type Mn-doped IV–VI^1^, III–V^2–4^, and II–VI^5,6^ compounds. Notably, the observation of a Curie temperature (*T*_C_) at 110 K in (Ga,Mn)As with a doping concentration of 5% Mn led to the pursuit of formation of DMSs with their *T*_C_ at or above room temperature (RT)^7^.

Inspired by the recent discovery of ferromagnetism in two-dimensional (2D) atomically thin layers, such as chromium triiodide (CrI_3_)^8,9^, chromium germanium telluride (Cr_2_Ge_2_Te_6_)^10^, and other van der Waals materials^11–13^, has moved the emphasis of studies from bulk crystals to low-dimensional materials. Transition metal dichalcogenide (TMD) monolayers, as atomically thin semiconductors, show unique thickness-dependent electrical and optical properties^14^ but remain nonmagnetic in their intrinsic form. Doping of transition metal elements, such as vanadium (V), Mn, and Fe, into TMDs to form atomically thin DMSs would permit the exploration of the magnetic coupling in 2D confined structures. Notably, first-principles studies predict that doping of transition metal ions into TMD monolayers is a promising way to realize a DMS with a *T*_C_ at or above RT^15,16^. Although being constrained by solubility and chemical stability, transition metal elements can be doped to some extent into monolayer TMDs, but ferromagnetism was not demonstrated^17–19^. Nevertheless, enhanced doping concentration of 2% Mn in Mn:MoS_2_ monolayers grown on a graphene substrate^17^ and 1% rhenium (Re) in Re:MoS_2_ monolayer exhibited suppression of defect-bound emission at low temperatures, demonstrating the feasibility of realizing in situ doping of TMD monolayers^20,21^. Employing a direct vapor-phase method, ferromagnetism was reported for Fe:SnS_2_ monolayer with a *T*_C_ of 31 K and a saturation magnetization of 3.5 emu g^−^^1^. However, in this approach, mechanical exfoliation is required after growing bulk crystals, which is not scalable. A recent study shows post-growth incorporation of V into MoTe_2_ monolayer with a small saturation magnetization of 0.6 µemu cm^−2^, giving rise to room-temperature ferromagnetism, but only in the form of small, randomly distributed clusters that are not uniform^21^. Another technique is based on ion implantation and the idea of co-doping, giving rise to very high saturation magnetization values of 60.62 emu g^−1^, but ferromagnetism is limited to a *T*_C_ around 10 K (ref. ^22^). These previous findings warrant a continued search for TMD-based DMSs that feature RT ferromagnetism and a scalable in situ growth technique that does not rely on detrimental post-processing techniques.

Here, we demonstrate successful in situ substitutional doping of Fe atoms into MoS_2_ monolayers directly via low-pressure chemical vapor deposition (LPCVD). We uncover an unambiguous Fe-related spectral feature in the luminescence of Fe:MoS_2_ monolayers, which is stable up to RT. The origin of the Fe-related luminescence peak is further supported by results obtained from a density functional theory (DFT) calculation of dipole-allowed transitions. In addition, we find that monolayer Fe:MoS_2_ displays ferromagnetism by probing the hysteresis in the magnetic circular dichroism (MCD) of the Fe-related emission. Moreover, by using nitrogen-vacancy (NV^–^) center magnetometry and magnetization measurement using superconducting quantum interference devices (SQUIDs), we provide clear evidence for RT ferromagnetism in our synthesized Fe:MoS_2_ monolayers and also quantitatively determine the local magnetic field.

## Results

### Synthesis and characterizations of Fe:MoS_2_ monolayers

**Fig. 1:**
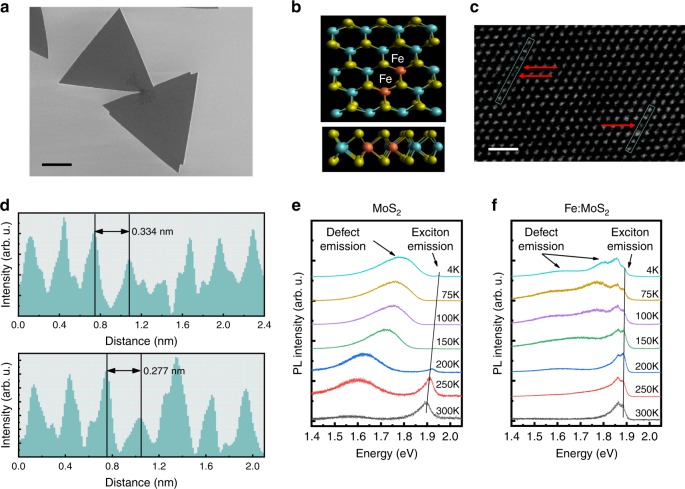
Fe:MoS_2_ monolayers. **a** SEM image of the as-grown Fe:MoS_2_ monolayer. Scale bar = 4 µm. **b** Schematic top and side view of the monolayer. The green, blue, and red spheres are sulfur, molybdenum and iron atoms, respectively. **c** Contrast-corrected STEM image of the as-grown Fe:MoS_2_ monolayer. Scale bar = 1 nm. **d** STEM intensity spectra of the selected area, where the Fe atom exhibits ∼40% lower intensity. Temperature-dependent PL from (**e**) as-grown MoS_2_ (**f**) Fe:MoS_2_. All PL spectra are taken under 100 µW pump power.

The in situ Fe doping of monolayer MoS_2_ was realized by growing MoS_2_ with Fe_3_O_4_ via the LPCVD contact-growth method^23^ (see Methods section). To eliminate the effects of local disorders in the substrate^24^, both as-grown MoS_2_ and Fe:MoS_2_ monolayers were encapsulated into thin-film hBN. Figure 1a shows a scanning electron microscopy (SEM) image of Fe:MoS_2_ monolayers. Triangular island-like domains were observed, which are typical for similar MoS_2_-CVD growth techniques. Figure 1b shows the schematic top and side views of the atomic structures of Fe:MoS_2_ monolayers. As substitution of Fe atoms at Mo sites is thermodynamically favorable^25^, Fe dopant atoms are shown in the schematic by replacing a single Mo host atom in the MoS_2_ crystal. To gain further insight into the atomic structure of the Fe:MoS_2_ monolayer, we employed high-angle annular dark-field scanning transmission electron microscopy (HAADF-STEM). Figure 1c shows the contrast-corrected STEM image of our Fe:MoS_2_ monolayer (original STEM image and related lattice distance measurements can be found in Supplementary Fig. 1). Compared with Mo (*Z* = 42) atoms, Fe (*Z* = 26) has ∼40% smaller atomic number. As the magnitude of the forward-scattered electron intensity is dependent on the atomic number, it is expected that Fe atoms produce lower relative intensity, which is clearly visible for the substitutionally doped Fe atoms in the STEM image. The corresponding STEM intensity scan in Fig. 1d verifies the intensity ratio of 0.38, which is consistent with the atomic number ratio and previously reported observations^26^. A broad Fe 2*p*3 peak from Fe:MoS_2_ monolayers was identified using X-ray photoelectron spectroscopy (XPS) and the atomic concentration was calculated as 0.3~0.5% (Supplementary Fig. 2). We observed a limited tunability in doping concentration in our synthesis, which is attributed to the high energy barrier to replace Mo by Fe^18^.

To verify the growth of monolayer Fe:MoS_2_ domains, we characterized the samples using atomic force microscopy (AFM). As depicted in Supplementary Fig. 3a, the AFM image occasionally shows the onset of the growth of the next layer, i.e., the bilayer of Fe:MoS_2_ with its typical snowflake-like pattern. The identification of bilayer growth is further evident from Supplementary Fig. 3b, which shows an identical step height thickness of 0.8 nm, commensurate with the thickness of Fe:MoS_2_ monolayers (0.8 nm) and bilayers (1.6 nm). The AFM images further confirm that, after wet cleaning and thermal annealing (see Methods section), the surface of Fe:MoS_2_ is free from any potential residual Fe_3_O_4_ particles from the doping process. Likewise, STEM images (Supplementary Fig. 4a, b) along with the energy-dispersive X-ray spectroscopy spectra (Supplementary Fig. 4c, d) also confirm that the surface of Fe:MoS_2_ monolayers is free of Fe_3_O_4_ particles.

### Optical analysis of Fe:MoS_2_ monolayers

Optical analysis via Raman and photoluminescence (PL) spectroscopy provides further evidence that Fe incorporates into the lattice (see Methods section). The Raman spectrum of Fe:MoS_2_ (Supplementary Fig. 5a) exhibits two typical characteristic vibration modes of MoS_2_ monolayers at *E*^1^_2g_ = 385.4 cm^−1^ (in-plane vibration of Mo and S atoms) and *A*_1g_ = 405.8 cm^−1^ (out-of-plane vibration of S atoms). Introducing the iron defects causes Raman linewidth broadening from 5.8 ± 0.1 to 7.6 ± 0.1 cm^−1^ for *A*_1g_ and from 4 ± 0.1 to 4.5 ± 0.1 cm^−1^ for *E*^1^_2g_. A previous study^27^ on oxygen-bombarded monolayer MoS_2_ shows a comparable broadening of MoS_2_ Raman modes due to a small lattice distortion caused by oxygen molecules. Distortion of the MoS_2_ lattice is also known to give rise to the optical quenching of the PL intensities^28^. To further investigate the lattice distortion, we compare the RT PL spectra of MoS_2_ and Fe:MoS_2_ monolayers shown in Supplementary Fig. 5b. The observed strong PL quenching is attributed to additional nonradiative recombination channels (trap states) that are introduced by the Fe doping, i.e., an indirect indicator that Fe-related defect states have been created. Evolution of PL intensity as a function of temperature for Fe:MoS_2_ and MoS_2_ monolayers are shown in Fig. 1e and Fig. 1f, respectively. A well-documented A-exciton peak at ∼1.92 eV was observed in MoS_2_ monolayers at 4 K. In addition, an abundant asymmetric peak at ∼1.8 eV was observed, which is caused by anion di-vacancies acting as point defects^29^. The dominance of the defect-induced exciton emission over the A exciton at 4 K suggests the presence of a large density of defects in CVD-grown MoS_2_ (ref. ^30^). By contrast, the Fe:MoS_2_ monolayer exhibits about the same intensities for the defect-induced emission and the A exciton, suggesting that Fe:MoS_2_ monolayers possess a lower native point defect density^29^, and thus a lower sulfur vacancy concentration. Previous studies show that transition metal doping of MoSe_2_ and MoS_2_ can suppress the Se or S vacancies^19,30^. The reduced sulfur vacancies in Fe:MoS_2_ monolayers can be caused by the Fe doping, which is evident from the Mo 3*d* electron peak in the XPS spectra (Supplementary Fig. 2b). The Mo-O bond peak intensity of Fe:MoS_2_ monolayers is lower than that of undoped MoS_2_ monolayers, suggesting a lower number of sulfur vacancies, commensurate with a recent study of Re-doped MoS_2_ monolayers^19^.

### Optical emission of Fe defect centers

**Fig. 2:**
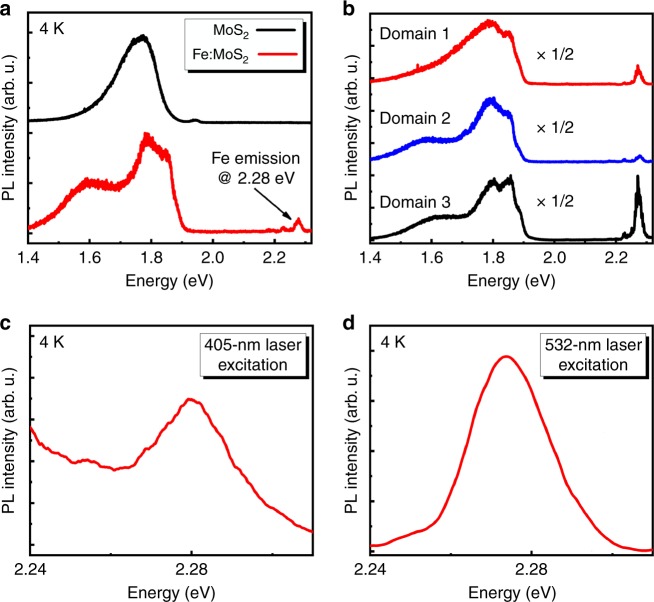
Fe-related spectral transition. **a** PL spectra of as-grown MoS_2_ (black) and Fe:MoS_2_ (red) measured at 4 K. The emergence of an emission peak at 2.28 eV in Fe:MoS_2_ is clearly visible. **b** PL spectra of Fe:MoS_2_ for three different domains revealing a high spatial sensitivity of the observed transition. For illustrative purpose, the Fe:MoS_2_ excitonic emission region below 2 eV is scaled down by a factor 2. **c**, **d** PL spectra of the 2.28 eV emission peak excited by 405 nm and 532 nm pump laser wavelength, respectively.

Interestingly, we also discovered a Fe-related radiative PL emission. Figure 2a shows the low-temperature PL emission of the Fe:MoS_2_ and MoS_2_ monolayers over a larger energy range, also including the above bandgap regime. When comparing the PL of Fe:MoS_2_ monolayers to that of MoS_2_, an emission peak at 2.28 eV becomes apparent. Figure 2b shows the Fe:MoS_2_ emission for three different triangles displaying large intensity variations of the 2.28 eV peak, which are likely caused by variations in the Fe dopant concentration between these differently oriented triangles. By contrast, PL mapping in Supplementary Fig. 6 reveals that both the defect luminescence and the Fe-related peak at 2.28 eV appears uniform within a given triangle. This result is expected, as the focused laser beam (spot size ~500 nm) covers several thousand Fe atoms at 0.5% Fe in the lattice, i.e., averages over any disorder in the Fe distribution on smaller length scales. The control experiments in Supplementary Fig. 7 also show that the Fe-related emission (2.28 eV) is only observed from Fe:MoS_2_ monolayers but not from undoped MoS_2_ with Fe or Fe_3_O_4_ clusters placed atop, indicating that only substitutional incorporation of Fe creates this transition. The thermal quenching behavior of the Fe-related emission peak is strikingly different from that of the exciton emission, which quenches with typical thermal activation energy around 30 meV, whereas the 2.28 eV transition is stable up to RT (Supplementary Fig. 8). This contrasting behavior is indicative of an underlying electronic transition at a defect state for the 2.28 eV emission. In addition, to rule out Fe-related local vibrational Raman modes as the origin of this transition, we recorded optical spectra in the vicinity of the 2.28 eV peak by detuning the wavelength of the excitation laser^31^ from 405 nm (Fig. 2c) to 532 nm (Fig. 2d). As can be seen, the observed peak position is unchanged, verifying that the observed Fe-related emission is not caused by a Raman vibrational transition mode, which would shift relative with the laser energy^19^. We thus attribute this feature to the PL emission of Fe defect centers^32,33^.

### DFT modeling of Fe-related emission

**Fig. 3:**
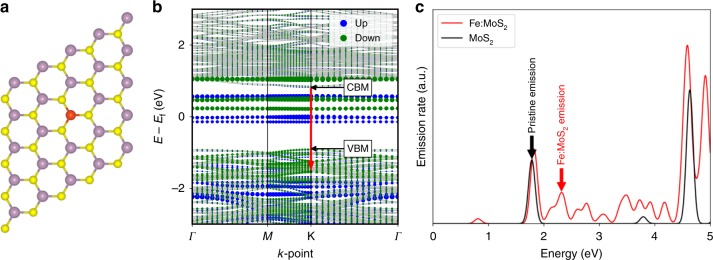
DFT calculations of dipole-allowed transitions in Fe:MoS_2_ monolayers. **a** 5 × 5 MoS_2_ supercell with a single Fe dopant used to simulate Fe:MoS_2_, where Mo, S, and Fe atoms are shown as purple, yellow, and red spheres, respectively. **b** Spin-polarized band structure of Fe:MoS_2_. Spin-up (down) states are shown in blue (green). The area of each circle is proportional to the projection of the state onto the Fe atom. The red arrow shows the transition corresponding to the peak labeled “Fe:MoS_2_ emission” in **c**. The CBM and VBM of the host MoS_2_ are also labelled. **c** Spontaneous emission rates of excited MoS_2_ and Fe:MoS_2_. The emission of both MoS_2_ and Fe:MoS_2_ was taken from the spin-up CBM states in their respective systems. For Fe:MoS_2_, this state is indicated by the tail of the red arrow in **b**.

To investigate the microscopic origin of the Fe-related PL peak at 2.28 eV, we used DFT to calculate the electronic structure of Fe:MoS_2_ (see Methods section). An isolated Fe dopant was simulated by replacing a single Mo atom with Fe in a 5 × 5 MoS_2_ supercell, as shown in Fig. 3a. Figure 3b plots the spin-polarized band structure for this system, where the area of each blue (green) circle is proportional to a spin-up (down) state’s overlap with a sphere of radius 1.3 Å centered on the Fe atom. The plot indicates that the presence of Fe introduces states that lie inside of MoS_2_’s pristine bandgap. In addition, the fact that the large blue and green circles inside the gap do not overlap indicates that the Fe induces a magnetic moment. Figure 3c plots the spontaneous emission (SE) rates from the spin-up conduction band minimum (CBM) state and compares them with those from the CBM in pristine MoS_2_. We see that the smallest emission energy for the pristine MoS_2_ state is ∼1.79 eV, corresponding to the large PL peak in Fig. 2a, which arises from relaxations across the pristine bandgap. The presence of Fe introduces another significant transition with an energy of ∼2.32 eV, which corresponds to the experimentally observed Fe:MoS_2_ emission PL peak at 2.28 eV. The expected magnitude of the PL peak is much smaller than what is shown in Fig. 3c, because any valence band hole left from the laser-excitation very quickly relaxes nonradiatively to the valence band maximum (VBM). Therefore, holes will spend very little time at the valence band state corresponding to the 2.28 eV transition, making this transition much less likely than the transition to the VBM.

### Magnetic characteristics of Fe:MoS_2_ monolayers

**Fig. 4:**
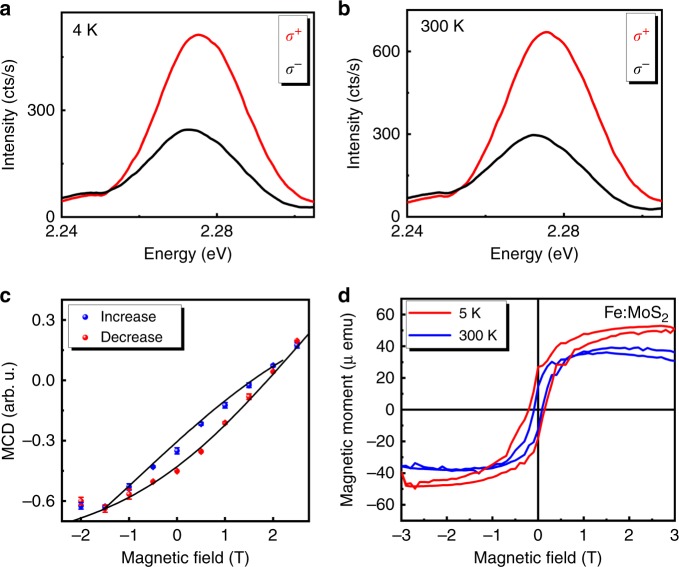
Magneto-photoluminescence measurements of Fe-related emission. **a**, **b** Fe-related spontaneous emission under excitation with opposite circularly polarized light states at 4 K and 300 K. The red and black spectra correspond to excitations with σ^+^ and σ^−^ polarizations, respectively. **c** Corresponding magnetic circular dichroism (MCD) as a function of increasing (blue circles) and decreasing (red circles) magnetic field, recorded at 4 K. **d** Magnetization data of Fe:MoS_2_ monolayers at cryogenic and room temperatures, after subtracting the diamagnetic background. A pronounced hysteresis loop is observed in Fe:MoS_2_ monolayers, suggesting ferromagnetism is preserved up to room temperature (300 K).

It is known that the optical emission from transition metal ion complexes usually originates from a charge transfer between the ligands and the transition metal^34^. The spin angular momentum of the electrons in an ion is sensitive to the handedness of polarization due to the spin selection rules of the circularly polarized light. Thus, transition metal ions show unequal amounts of light absorption when excited with left- and right-handed circular polarizations^35^. At the atomic level, the light absorption is closely related to the magnetically induced Zeeman shifts. Therefore, performing MCD spectroscopy can give insights into the magnetic properties of the material. Figure 4a, b show the Fe-related PL spectra of Fe:MoS_2_ under excitations with opposite circularly polarized light at both 4 K and RT. Circular dichroism (CD) was determined using the well-known relation $\rho = \frac{{I_ + -\, I_ - }}{{I_ + \,+\, I_ - }}$, where $I_ \pm$ are the PL intensities with left- and right-handed polarized excitations. The Fe-related emission shows a strong CD (*ρ* ≈ 40%) at both 4 K and RT. Given that the transition metals’ luminescence loses its CD above the Curie temperature *T*_C_^36^, observation of a strong CD at 300 K suggests that Fe:MoS_2_ remains ferromagnetic at RT. To verify the existence of the ferromagnetic phase in Fe:MoS_2_, the ferromagnetic hysteresis was investigated by analyzing the CD components as a function of an external magnetic field. Figure 4c shows the MCD of the Fe-related emission as a function of increasing (blue circles) and decreasing (red circles) magnetic field ranging from −3 T to 3 T at 4 K. The pronounced hysteresis loop clearly identifies the ferromagnetic nature of the Fe-related PL emission. As the recording of MCD data is limited to cryogenic temperatures in our magneto-PL system, we have carried out magnetization measurements with a SQUID magnetometer (see Methods section). Figure 4d shows that Fe:MoS_2_ monolayers exhibit a pronounced *M*–*H* hysteresis loop at both cryogenic (5 K) and RT, corroborating that our synthesized Fe:MoS_2_ monolayers display ferromagnetism even at 300 K. The magnitude of the hysteresis loop decreases with increasing temperature due to the decreasing coercive field under thermal load, whereas the *T*_C_ has not been reached at 300 K. The *M*–*H* curve at 300 K shows nonlinearity and small hysteresis up to 3 T, attributed to superparamagnetic and weakly ferromagnetic regions in the sample. The bifurcation of the curves at 3 T is within the error of our measurement, as the *M*–*H* curves are obtained after subtracting the linear diamagnetic background, which is well known in TMD semiconductors^37^.

### Local magnetometry of Fe:MoS_2_ monolayers

To further quantify the local strength of the ferromagnetic field at RT in Fe:MoS_2_, we performed magnetometry using NV^−^ centers in nanodiamonds. This technique relies on the optically detected magnetic resonance (ODMR) of the electron spins of NV^−^ centers manipulated by simultaneous microwave (MW) radiation. Such NV^−^ spin systems, which are readily accessible by visible lasers and radiation, are important quantum resources for quantum information and nanoscale sensing of temperature^38^, magnetic field^39^, and beyond^40^. We analyze the relative Zeeman shifts in the electron spin resonance (ESR) of NV^−^ centers’ ground state sublevels to map out the magnitude of the local magnetic field induced by the Fe atoms in our Fe:MoS_2_ samples (see Methods section). Due to the short-range nature of the magnetic field induced by a single transition metal atom, it is crucial to proximity couple^41^ the sensing NV^−^ centers to the sample. To maintain a short spacing between the NV^−^ centers and the sample, the NV^−^ centers were spin-coated on the surface of Fe:MoS_2_ monolayers.

**Fig. 5:**
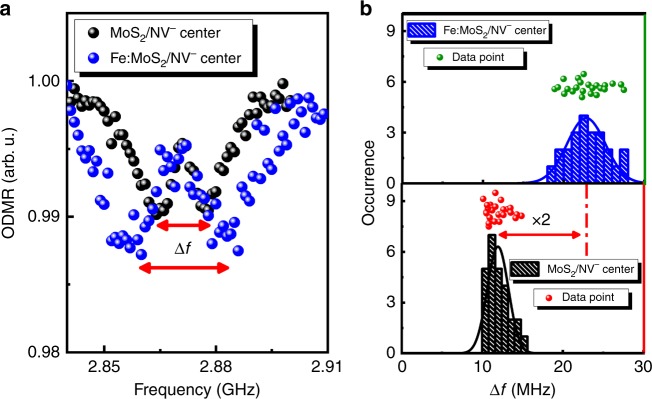
Local magnetic field of Fe:MoS_2_ monolayers. **a** Comparison of ODMR spectrum of the NV^−^ centers coated on as-grown MoS_2_ and Fe:MoS_2_ monolayers, respectively, demonstrating additional Zeeman splitting due to Fe:MoS_2_ local magnetic field. **b** Corresponding occurrence histograms for NV^−^ centers frequency splitting between the two spin sublevels, recorded at room temperature.

An exemplary ODMR spectrum recorded for the Fe:MoS_2_ and MoS_2_ is shown in Fig. 5a. The energy splitting between the two typical ESR dips of the NV^−^ centers was found to be ~21 MHz in the vicinity of Fe:MoS_2_ monolayers. This splitting was significantly larger than that for undoped MoS_2_ (~10 MHz) caused by the pseudo-magnetic field of nanodiamonds, indicating the presence of a local magnetic field in Fe:MoS_2_ monolayers. Figure 5b shows the histogram of Zeeman energy splittings recorded for 24 and 20 different positions on Fe:MoS_2_ and undoped MoS_2_ monolayers, respectively. Statistical analysis shows that the mean Zeeman energy splitting on Fe:MoS_2_ has increased by ~11 MHz compared with bare MoS_2_. This difference was analyzed to determine the mean local magnetic field using the Zeeman splitting term $\Delta E = g_{\mathrm{e}}\mu _{\mathrm{B}}{\mathbf{B}} \cdot \hat S$ in the NV^−^ center’s spin Hamiltonian^40^. In this equation, the change in the Zeeman splitting is related to the projection of a magnetic field on the NV^−^ center’s spin, and $g_{\mathrm{e}}$ and $\mu _{\mathrm{B}}$ are the Landé *g*-factor and Bohr magneton, respectively. We found that the sample’s local magnetic field can be as large as 0.5 ± 0.1 mT. This value is close to that measured in the 2D CrI_3_ and CrBr_3_ ferromagnets at cryogenic temperature^42,43^. The fact that Fe:MoS_2_ shows a large local magnetic field at RT is clear evidence that this material has preserved the magnetization induced on it upon loading. Therefore, it can be inferred from the data that the Fe:MoS_2_ monolayers with substitutionally incorporated Fe atoms act as a DMS displaying RT ferromagnetism.

## Discussions

We have demonstrated in situ substitutional doping of Fe atoms in MoS_2_ monolayers via LPCVD. The presence of Fe atoms in the MoS_2_ lattice was verified using STEM and Raman spectroscopy. PL spectroscopy revealed an unambiguous Fe-related emission at 2.28 eV in Fe:MoS_2_ monolayers, which is stable up to RT. This observation was rationalized by DFT calculation of dipole-allowed transition rates. In addition, we demonstrated that monolayer Fe:MoS_2_ displays ferromagnetism by probing the hysteresis of the MCD of the Fe-related emission at 4 K. As a key finding, studies involving NV^−^ center magnetometry show that Fe:MoS_2_ monolayers remain magnetized, with a local field up to 0.5 ± 0.1 mT even at ambient conditions, manifesting ferromagnetism at RT for this material. These findings extend the class of available ferromagnetic van der Waals materials with ferromagnetism at or above RT and open opportunities towards applications such as on-chip magnetic manipulation in quantum information science or in minimizing bit storage in spintronics.

## Methods

### Synthesis of MoS_2_ and Fe-doped MoS_2_ monolayers

MoS_2_ monolayers were synthesized via LPCVD^23,44^. Prior to growth, a thin MoO_3_ layer was prepared using physical vapor deposition (PVD) of MoO_3_ onto a Si substrate with 300 nm-thick thermal oxides. Another SiO_2_/Si substrate contacted the MoO_3_-deposited substrate face-to-face. Fe:MoS_2_ monolayers were grown onto the SiO_2_/Si substrate. The Fe doping was achieved in the following sequence: Fe_3_O_4_ particles were evenly cast onto the SiO_2_/Si substrate before contacting the MoO_3_-deposited substrate. To ensure a uniform distribution of Fe_3_O_4_ particles, the substrate was washed using deionized (DI) water, so that a thin layer of water was created on the SiO_2_ surface^45^, prior to applying the Fe_3_O_4_ particles. The substrate was then annealed at 110 °C for 5 min on a hot plate. For the growth, the furnace was heated up with a ramping rate of 18 °C min^−1^ and held for 15 min at 850 °C. During the heating procedure, an argon gas (30 s.c.c.m.) was supplied at 300 °C and, subsequently, a hydrogen gas (15 s.c.c.m. of) was delivered at 760 °C. Sulfur was supplied when the furnace temperature reached 790 °C. After the growth, a few millimeters size of Fe:MoS_2_ monolayers were obtained.

### Sample preparation for characterization

Multilayers hBN (13 nm thick) were grown via CVD on a copper foil with a borazine precursor (Graphene Supermarket). To transfer the multilayers hBN onto the silicon substrate, polymethyl methacrylate (PMMA) in chlorobenzene (950 C4) was spin-coated (200 rpm) onto the multilayers hBN and baked at 60 °C for 5 min. Samples were placed in copper etchant (Transene) at 100 °C for 12 h to dissolve the copper foil. The resultant multilayers hBN/PMMA films were transferred onto the 300 nm SiO_2_/Si substrate. As-grown MoS_2_ and Fe:MoS_2_ on SiO_2_ were coated with a thin layer of PMMA 950 A4 using a dropper, then left in air at RT for 2 h to drive off the solvent. The chips were floated in 30% KOH (aq); after 10–40 min the Si chip falls to the bottom leaving the PMMA + MoS_2_ square floating on the surface. The PMMA was cleaned in filtered DI water and blow-dried with RT air. The PMMA + MoS_2_ was successfully transferred to lacey carbon TEM grids or hBN/SiO_2_/Si substrates by placing the MoS_2_ side down and removing PMMA by warm acetone at 60 °C for 30 min. To remove the PMMA stabilization layer, samples were first rinsed with warm acetone and then annealed in an ultrahigh vacuum chamber on top of a button heater (Heatwave Laboratories) for 4 h (MoS_2_ and Fe:MoS_2_) or 12 h (hBN) at 350 °C to fully remove any residual polymer contamination.

### Microscope characterizations

SEM observations were carried out using Zeiss Auriga Small Dual-Beam FIB-SEM in the Laboratory for Multiscale Imaging (LMSI) at Stevens Institute of Technology. This microscope has a GEMINI field-emission electron column that provides a resolution of 1.0 nm @ 15 kV and 1.9 nm @ 1 kV. The SEM images were acquired at 2 kV with the in-lens EsB (energy selective backscattering) detector. HAADF-STEM observations were carried out using a probe-corrected Hitachi HD 2700C at the Center for Functional Nanomaterials at Brookhaven National Laboratory. The microscope has a cold field-emission gun source. The STEM images were acquired at 200 kV with a spatial resolution of 1.4 Å. AFM observations were carried out by Bruker MultiMode 8 AFM at the Advanced Science Research Center at the City University of New York (CUNY).

### XPS characterization

Both doped and undoped samples were analyzed using XPS (VersaProbe II from Physical Electronics). All the measurements were performed with Al Kα X-rays, the 45° measuring angle, under low-energy electron and Ar^+^ surface charge neutralization, with 11.74 eV pass energy for individual peaks and 117.40 eV for survey spectrum. The binding energy of 284.8 eV for the main C1s (C-C/C-H) was used to correct of charging of specimen under irradiator. The spectral analysis software Multipak was employed for XPS peak deconvolution, where Voigt line shape and an active Shirley background were used for the peak fitting. The atomic doping concentration was calculated by determining the area ratio of the integrated peak intensities of Fe 2*p*3, Mo 3*d*, and S 2*p* peaks of Fe:MoS_2_ monolayers.

### Optical measurements

Micro-PL measurements were taken inside an ultra-low vibration cryogen-free cooling closed-cycle cryostat (attoDRY1100) with a temperature range from 4 K to 300 K. The magneto-PL of the sample was studied at 4 K in the Faraday geometry. For optical excitation, we utilized nonresonant laser pumping below the bandgap (2.4 eV). Circularly polarized laser pumping was achieved by passing the laser beam through a linear polarizer followed by a zero-order quarter-wave plate. The reflected PL signal was collected by the same objective and guided by a multimode fiber to a spectrometer with an attached liquid-nitrogen-cooled silicon charge-coupled device camera. Magnetic fields were applied perpendicular to the plane of the sample within the range −3 to 3 T.

### SQUID measurement

Magnetization measurements were performed in a commercial R-700X SQUID magnetometer from Cryogenic Limited. As-grown Fe:MoS_2_ monolayers on Si/SiO_2_ substrates were diced to 3 × 3 mm^2^ chips and loaded into a gel capsule. The magnetic moment was measured at 5 K and 300 K, while cycling a DC magnetic field through −3 T to 3 T.

### NV^−^ center magnetometry

The orbital ground state in the negatively charged NV^−^ centers splits into a triplet state ($m_{\mathrm{s}} = 0, \pm\! 1$). In a degenerate triplet state, the $m_{\mathrm{s}} = 0$ state is separated by a ~2.87 GHz from $m_{\mathrm{s}} = 1, - 1$. Readout of the spin state within the ground state triplet can be performed optically using the spin-selective intersystem crossing between the NV^−^ centers excited state triplet and a pair of singlet states. Decay through the intersystem crossing is nonradiative, and preferentially repopulates the $m_{\mathrm{s}} = 0$ ground state spin sublevel. Thus, it generates a reduction in the emitted PL results if the NV^−^ centers spin has been prepared in either of its $m_{\mathrm{s}} = - 1$ or $m_{\mathrm{s}} = + 1$ sublevels using MW radiation prior to optical excitation. The spin states degeneracy ($m_{\mathrm{s}} = {\pm}\! 1$) can be lifted by a magnetic field due to the Zeeman effect, leading to the appearance of two resonance dips in the ODMR spectrum. In nanodiamonds, the crystal lattice is typically strained, generating a pseudo-magnetic field that gives rise to a broken degeneracy even in the absence of an external magnetic field. Thus, the energy splitting in the Fe:MoS_2_ ODMR signal can be calibrated to measure the sample’s local magnetic field.

The ODMR-based magnetometry was performed in a confocal microscopy setup. The NV^−^ centers were excited using a frequency-doubled Nd:YAG laser (532 nm, 300 µW) focused on the sample with a NA = 0.8 objective. The fluorescence signal of the NV^−^ centers was then collected by the same objective and spectrally filtered for ODMR measurements. To obtain the ODMR signal, the electron spin states were excited $m_{\mathrm{s}} = 0 \leftrightarrow m_{\mathrm{s}} = {\pm}\! 1$ by MW pulses. We employed a loop antenna centered about the sample to deliver the required MW intensity to the sample. For a given frequency (in the range of 2.84~2.91 GHz), the antenna delivers a sequence of 100 ms alternative on/off MW pulses to control the electron spin of NV^−^ centers. The normalized PL is the ratio of total PL counts with and without MW excitation, where the resonance peaks occur at the frequencies of minimum ratio. Magnetic strength can be calculated by measuring the splitting frequency in the NV^−^ centers’ ODMR due to the sample’s local magnetic field.

### Computational methods

DFT^46,47^ calculations were carried out using the projector augmented wave^48,49^ method as implemented in the Vienna ab initio simulation package^50^. We employed the Perdew–Burke–Ernserhof generalized gradient approximation for the exchange-correlation functional^51^ with a basis set including plane waves with energies up to 600 eV. The Brillouin zone of pristine MoS_2_ was sampled with a 6 × 6 × 1 ${\mathrm{\Gamma }}$-centered Monkhorst-Pack grid^52^. Relaxation iterations continued until the Hellmann-Feynman forces on all atoms settled below 1 meV per Å, whereas electron field iterations persisted until changes in both the total energy and Kohn-Sham eigenvalues fell below 10^−^^7^ eV. Meanwhile, 20 Å of vacuum was inserted in the *z*-direction (out-of-plane direction) to negate interactions with the periodic images. Lastly, Fe:MoS_2_ was simulated by substituting a Mo atom with Fe in a 5 × 5 supercell, as shown in Fig. 3a. To explain the origin of the PL peak at 2.28 eV, it is imperative to show that SE from the CBM has a significant transition amplitude to a valence state about 2.28 eV below the CBM. To rationalize this process, we calculated the rate of SE from an initial state $|i\rangle$ to a final state $|f\rangle$, using^53^

1 $$A\left( {\omega _{fi}} \right) = \frac{{4\alpha }}{{3c^2}}\omega _{fi}^3\left|\langle {f|\hat r|i} \rangle\right|^2$$

where $\hbar \omega _{fi}$ is the energy difference between the states, *c* is the speed of light, *α* is the fine structure constant, and ${\hat{\mathbf{r}}}$ is the position operator. As the emitted photons carry very little momentum, we assumed that a relaxing electron’s crystal momentum does not change during the emission process. We also assumed that the electron retains its spin. Thus, we calculated the position matrix element between the CBM for both spin directions and every state of lower energy lying on the same *k*-point and possessing the same spin. Plugging these elements into Eq. (1) then gave the SE rates of all possible transitions from the CBM. Transitions from the spin-up CBM are plotted in Fig. 3c.

## Supplementary information

Supplementary Information

## Data Availability

The source data underlying Figs. 1e, f, 2a–d, 4a–d and 5a, b, and Supplementary Figs. 2a–c, 3b, 4c, d, 5a, b, 6a, 7, and 8a, b are provided as a Source Data file.

## Source Data

Source Data
