# Supplementary material for: Enabling room temperature ferromagnetism in monolayer MoS2 via in situ iron-doping
Source: Nat Commun. 2020 Apr 27;11:2034. doi: 10.1038/s41467-020-15877-7 (PMC7184740; doi:10.1038/s41467-020-15877-7)
Supplement: Supplementary file 1 — Supplementary Information [file 41467_2020_15877_MOESM1_ESM.pdf]

Supplementary Information

Enabling room temperature ferromagnetism in monolayer MoS_2 via *in situ* iron-doping

Shichen Fu[†,1], Kyungnam Kang[†,1], Kamran Shayan[†,2,3,4], Anthony Yoshimura[5], Siamak Dadras[4], Xiaotian Wang[1], Lihua Zhang[6], Siwei Chen[1], Na Liu[2,3], Apoorv Jindal[7], Xiangzhi Li[2,3], Abhay N. Pasupathy[7], A. Nick Vamivakas[4], Vincent Meunier[5], Stefan Strauf*[,2,3], and Eui-Hyeok Yang*[,1,3]

[†]These authors contributed equally to this work.

*Address correspondence to: eyang@stevens.edu, sstrauf@stevens.edu

[1]Department of Mechanical Engineering, Stevens Institute of Technology, Hoboken, New Jersey 07030, United States.

[2]Department of Physics, Stevens Institute of Technology, Hoboken, New Jersey 07030, United States.

[3]Center for Quantum Science and Engineering, Stevens Institute of Technology, Hoboken, New Jersey 07030, United States

[4]Institute of Optics, University of Rochester, Rochester, New York 14627, United States.

[5]Department of Physics, Applied Physics, and Astronomy, Rensselaer Polytechnic Institute, Troy, New York 12180, United States

[6]Center of Functional Nanomaterials, Brookhaven National Laboratory, Upton, New York 11973-5000, United States.

[7]Department of Physics, Columbia University, New York, New York 10027, United States

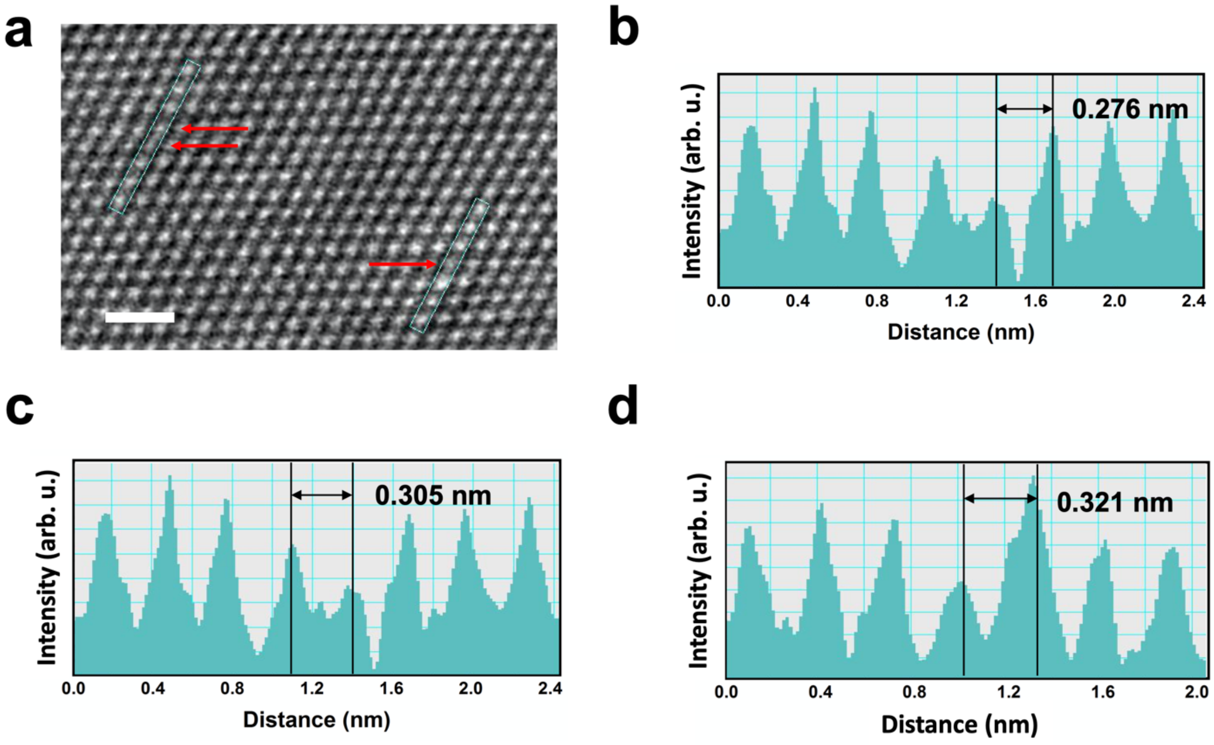

Supplementary Figure 1 (a) HAADF-STEM image of Fe:MoS_2 monolayer. Scale bar = 1 nm. **(b)** and **(c)** STEM intensity spectra of the selected area on the left in (a), **(d)** STEM intensity spectra of the selected area on the right in (a). Fe atom exhibits approximately 40% lower intensity.

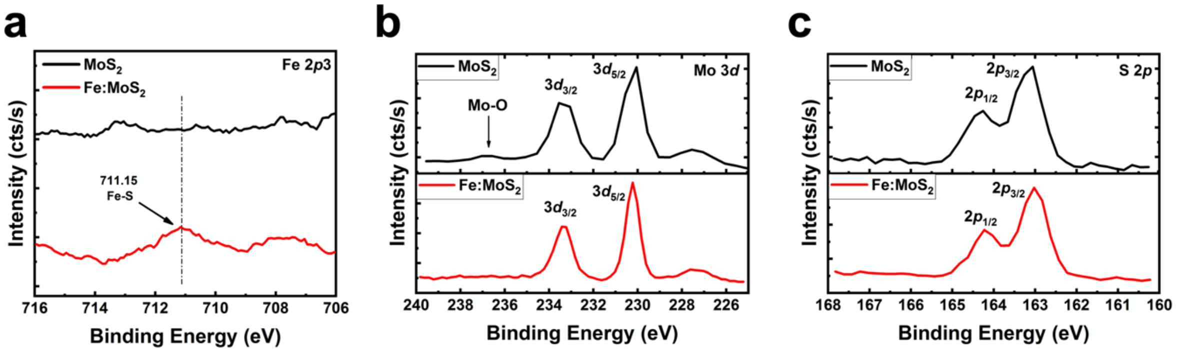

Supplementary Figure 2 X-ray photoelectron spectroscopy (XPS) of MoS_2 and Fe:MoS_2 monolayers. **(a)** Fe 2*p*3 peak in Fe:MoS_2 monolayers. **(b)** Mo 3*d* peak. The reduced intensity of the Mo-O bond in Fe:MoS_2 (237 eV) as compared to MoS_2 is indicative of an underlying reduction of the sulfur vacancy concentration upon doping. **(c)** Corresponding S 2*p* peak signal.

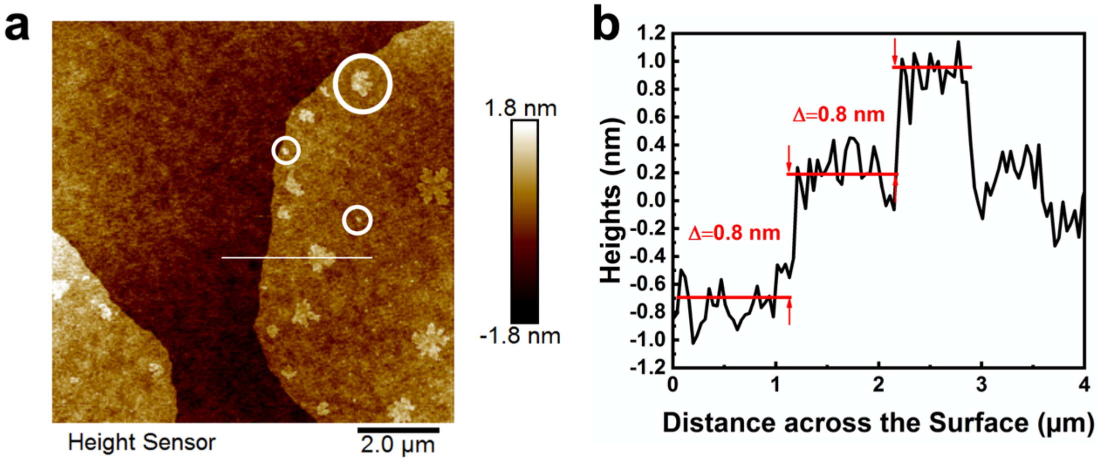

Supplementary Figure 3. (a) Atomic force microscope (AFM) topographical image of Fe:MoS_2. Bilayers or bilayer nuclei are indicated by white circles. **(b)** Corresponding AFM line scanning across the line (white line) drawn in (a). This shows a 0.8 nm thickness of Fe:MoS_2 monolayer, confirming the formation of monolayers.

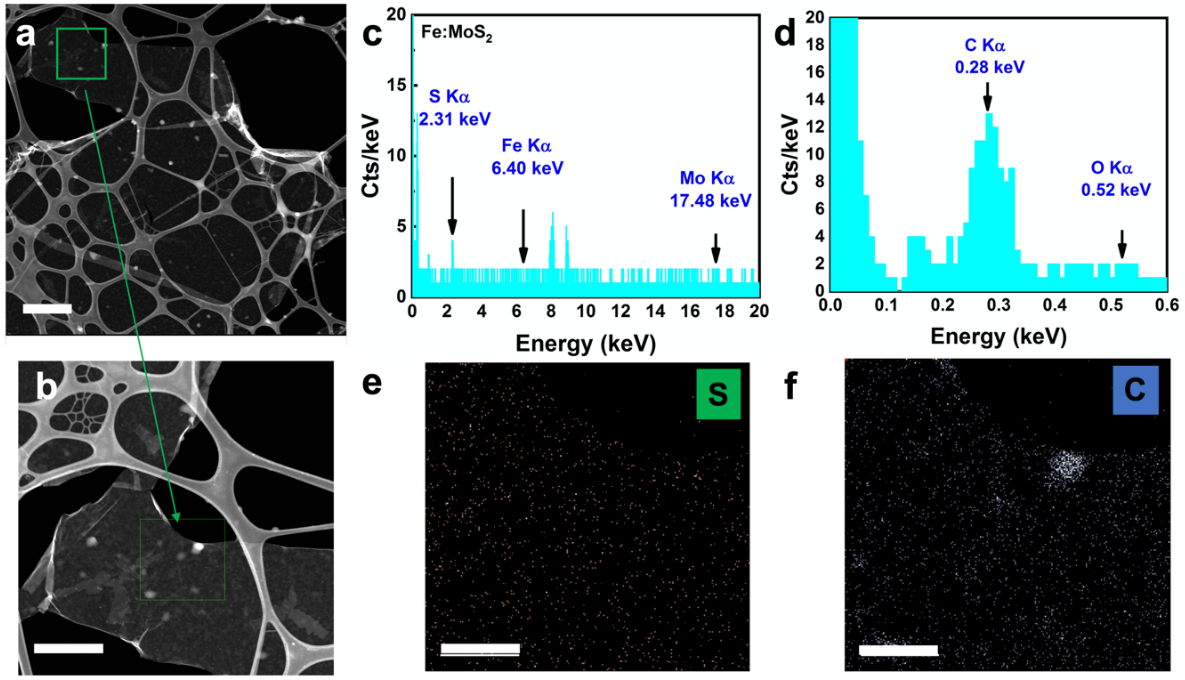

Supplementary Figure 4. STEM image (left) and EDS mapping (center and right) of Fe:MoS_2 monolayers. Scale bar = 1 μm. **(a)** Low-magnification STEM image of Fe:MoS_2 monolayers. Scale bar = 700 nm. **(b)** STEM image of EDS mapped region (800 nm x 800 nm). EDS spectra of the mapped area with **(c)** 0 to 20 keV and **(d)** 0 to 0.6 keV. **(e, f)** EDS mapping of S and C within the green square area in (b). Scale bar = 200 nm. C signal matches the “bright spot” shown in the green square area in (b), showing that such bright spots are polymer residues left after layer transfer to the TEM grid.

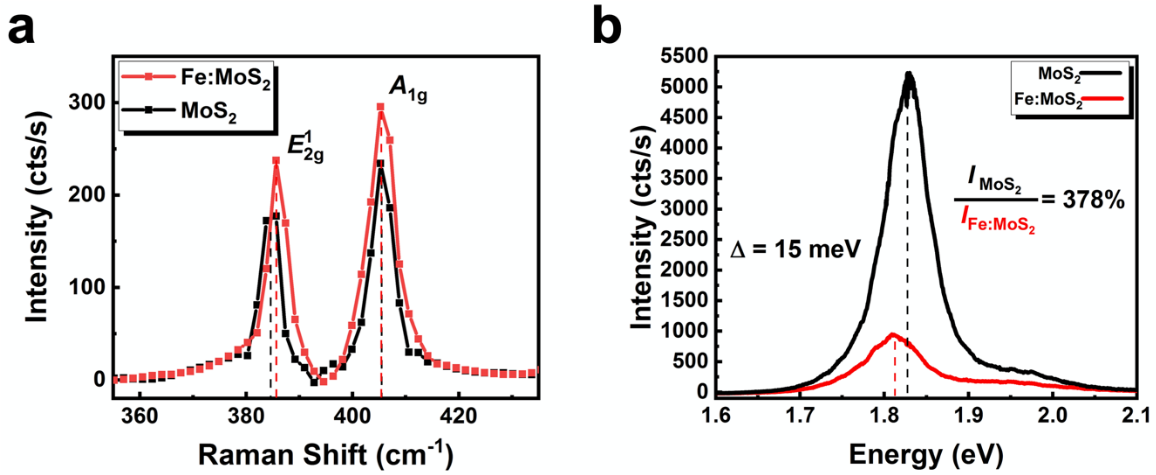

Supplementary Figure 5 (a) Raman spectra and (**b**) PL spectra comparing MoS_2 and Fe:MoS_2 monolayers. All data were taken at room temperature.

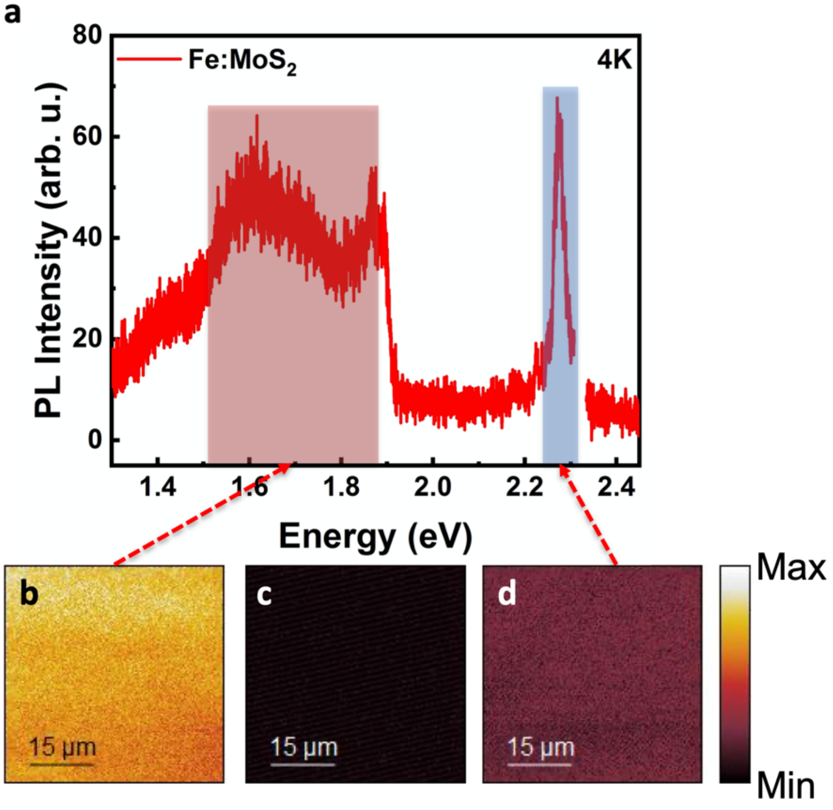

Supplementary Figure 6. PL mapping of Fe:MoS_2. **(a)** PL spectrum of Fe:MoS_2 monolayers recorded at 4K. **(b)** Hyperspectral PL scan of defect emission filtered over 650 nm to 800 nm (red shading). **(c)** Background signal from off-sample location. **(d)** Hyperspectral PL scan of Fe-related emission filtered over 540 nm to 560 nm (blue shading). Data have been collected with high numerical aperture objective (NA=0.83) resulting in a near resolution limited spot size of about 0.5 µm and scanned over an area of 40 µm x 40 µm.

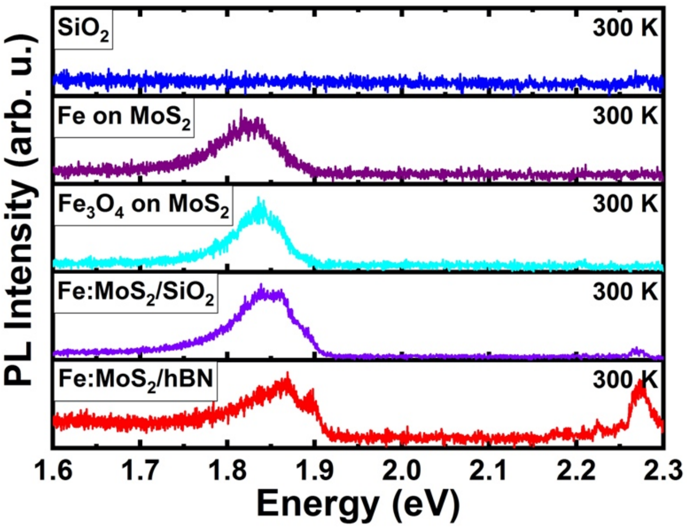

Supplementary Figure 7. PL spectra of control samples at 300K. Fe-related emission (2.28 eV) is only observed from Fe:MoS_2 monolayers but not from undoped MoS_2 where either Fe or Fe_3O_4 clusters where placed atop, indicating that only substitutional incorporation of Fe creates this transition.

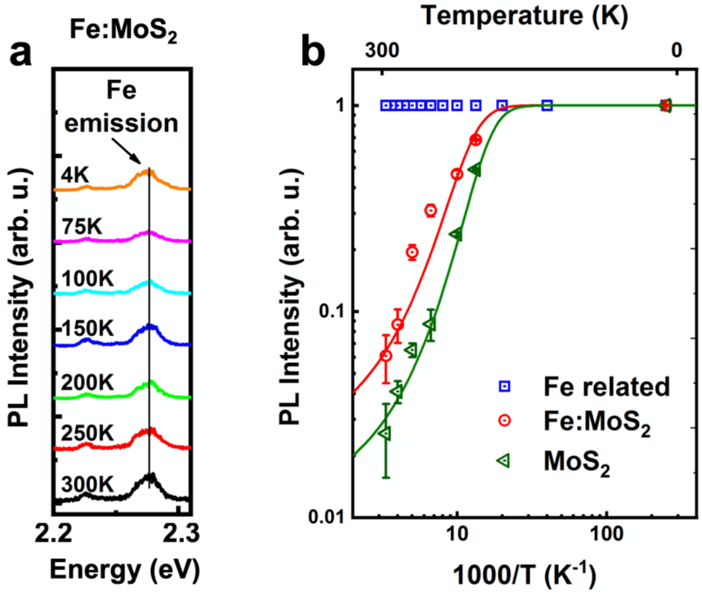

Supplementary Figure 8 (a) Temperature dependent PL spectra of Fe related emission around 2.28 eV. **(b)** Arrhenius plot of integrated PL for the bandgap emission in MoS_2 (green triangle), Fe:MoS_2 (red circle) and for the Fe related emission at 2.28 eV (blue square). The solid red and green lines are standard Arrhenius fits using a thermal activation energy E_A for the exciton emission (sum of free and bound exciton peaks) resulting in 28±2 meV for MoS_2 and 32±2 meV for Fe:MoS_2 monolayers. All PL spectra are taken under 100 μW pump power.
